# Visualising the 3D microstructure of stained and native intervertebral discs using X-ray microtomography

**DOI:** 10.1038/s41598-017-16354-w

**Published:** 2017-11-24

**Authors:** C. M. Disney, K. Madi, A. J. Bodey, P. D. Lee, J. A. Hoyland, M. J. Sherratt

**Affiliations:** 1 Centre for Doctoral Training in Regenerative Medicine, University of Manchester, Manchester, UK; 2 Division of Cell Matrix Biology and Regenerative Medicine, University of Manchester, Manchester, UK; 3 School of Materials, University of Manchester, Manchester, UK; 4 Diamond Light Source, Harwell Science and Innovation Campus, Oxfordshire, UK; 5 NIHR Manchester Biomedical Research Centre, Central Manchester University Hospitals NHS Foundation Trust, Manchester Academic Health Science Centre, Manchester, UK

## Abstract

Intervertebral disc degeneration (IVDD) is linked to low back pain. Microstructural changes during degeneration have previously been imaged using 2D sectioning techniques and 3D methods which are limited to small specimens and prone to inducing artefacts from sample preparation. This study explores micro computed X-ray tomography (microCT) methods with the aim of resolving IVD 3D microstructure whilst minimising sample preparation artefacts. Low X-ray absorption contrast in non-mineralised tissue can be enhanced using staining and phase contrast techniques. A step-wise approach, including comparing three stains, was used to develop microCT for bovine tail IVD using laboratory and synchrotron sources. Staining successfully contrasted collagenous structures; however not all regions were stained and the procedure induced macroscopic structural changes. Phase contrast microCT of chemically fixed yet unstained samples resolved the nucleus pulposus, annulus fibrosus and constituent lamellae, and finer structures including collagen bundles and cross-bridges. Using the same imaging methods native tissue scans were of slightly lower contrast but free from sample processing artefacts. In the future these methods may be used to characterise structural remodelling in soft (non-calcified) tissues and to conduct *in situ* studies of native loaded tissues and constructs to characterise their 3D mechanical properties.

## Introduction

**Figure 1:**
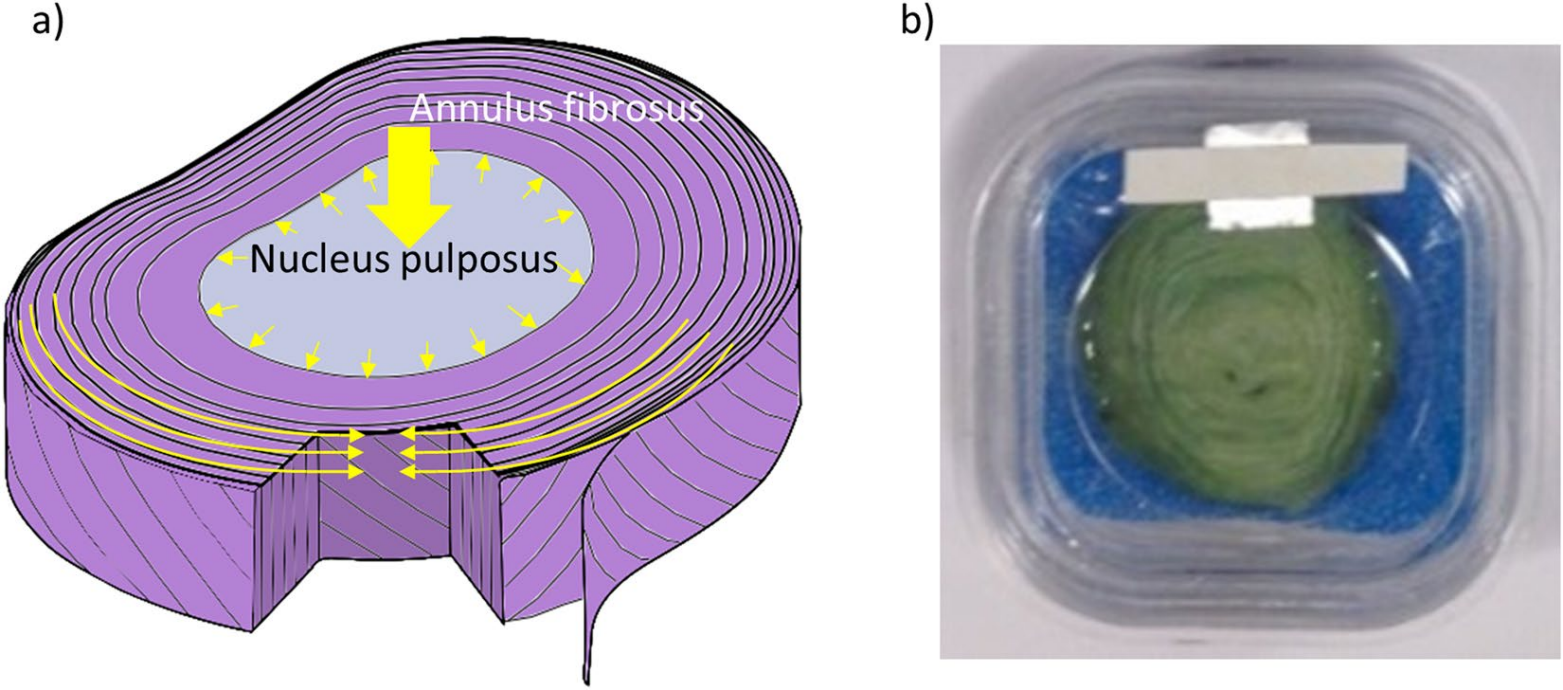
Anatomy of the intervertebral disc. Schematic of the IVD (**a**). IVDs are situated between the vertebral bodies in the spine. They provide flexibility and bear load caused by body weight and physical activity. A portion is cut out to show the central gelatinous NP and the alternating orientation of collagen bundles between adjacent AF lamellae. Yellow arrows indicate the load path. Bovine tail intervertebral disc stained with PMA and stored in membrane box for scanning (**b**).

Low back pain (LBP) affects up to 84% of the population at some point during their lives^1^. Although the aetiology of LBP is complicated, in 40% of cases irreversible structural degeneration of the intervertebral disc (IVD) is thought to be responsible^2^. At the centre of each disc is the proteoglycan-rich nucleus pulposus (NP) which is hydrophilic due to a high negative charge density and hence osmotic pressure^3^. The NP is surrounded by the annulus fibrosus (AF), an outer circumferential ring of fibrocartilage whose anisotropic mechanical behaviour is determined by concentric lamellae composed of alternately angled collagen I fibril bundles^4–6^ (Fig. 1a). There are many factors linked to IVD degeneration such as ageing^2,7^, genetics^8,9^, reduced nutrient supply and abnormal loading which, as a result of altered cellular and molecular events, causes morphological changes including loss of NP height^10,11^ and increasingly disorganised AF structure^12,13^. The initiation of the degeneration process has been found to have distinct phenotypes which relate to different risk factors^14,15^. In order therefore, to characterise the complex nature of IVD structural degeneration and the *in situ* mechanical competency of tissue-engineered IVD replacements, it will be necessary to develop new analysis methods which are capable of visualising and quantifying the effects of applied load on the 3D microstructure of native tissues and organs.

Previously IVD micro-structure has been characterised by 2D imaging of either tissue sections or the surface of sequentially peeled AF lamellae^4,5,16–19^. However these approaches are: (i) destructive and prone to inducing artefacts, (ii) disruptive of the large residual strains which characterise the unloaded IVD state^20^, (iii) reliant on chemical fixation or partial dehydration of the tissues which alters their structure and mechanical properties^21,22^ and (iv) confined to imaging relatively small regions of the disc. X-ray micro-computed tomography (microCT) has the potential to circumvent these issues by imaging native (non-chemically fixed) tissues in 3D at microscopic resolutions.

Soft tissues and their constituent components weakly attenuate X-rays and as a consequence many microCT imaging studies rely on the use of heavy metal stains to non-specifically enhance X-ray contrast in whole organisms or multiple organs^23–28^. Phosphomolybdic acid has been shown to have high affinity for collagens, for example characterising collagen 3D distribution in single organs or tissues at histological resolutions^29–31^. However, in most cases these staining procedures are poorly characterised with regards to their biochemical specificity, commonly require the use of chemical fixatives and struggle to penetrate large tissue volumes. An alternative phase-contrast approach is needed to image relatively large native tissues and organs at microscopic resolutions. In-line propagation-based phase contrast imaging makes use of varied refraction of the X-ray wave-front as it passes through an object of varied refractive index. This gives rise to interference fringes and contrast enhancement at the edges of structures and has been shown to be applicable to biomedical applications in which absorption contrast between structures is weak^32–34^.

This study uses a step-wise approach encompassing stained, chemically fixed and native tissues imaged by microCT on ‘laboratory’ (microfocus tube) and synchrotron X-ray sources with the aim of minimising sample interaction or the effect of preparation on native structure. In the longer term, these methods may be used to characterise age-related structural remodelling in cartilaginous tissues and to map the 3D mechanical properties of tissues and tissue-engineered constructs.

## Results

**Figure 2:**
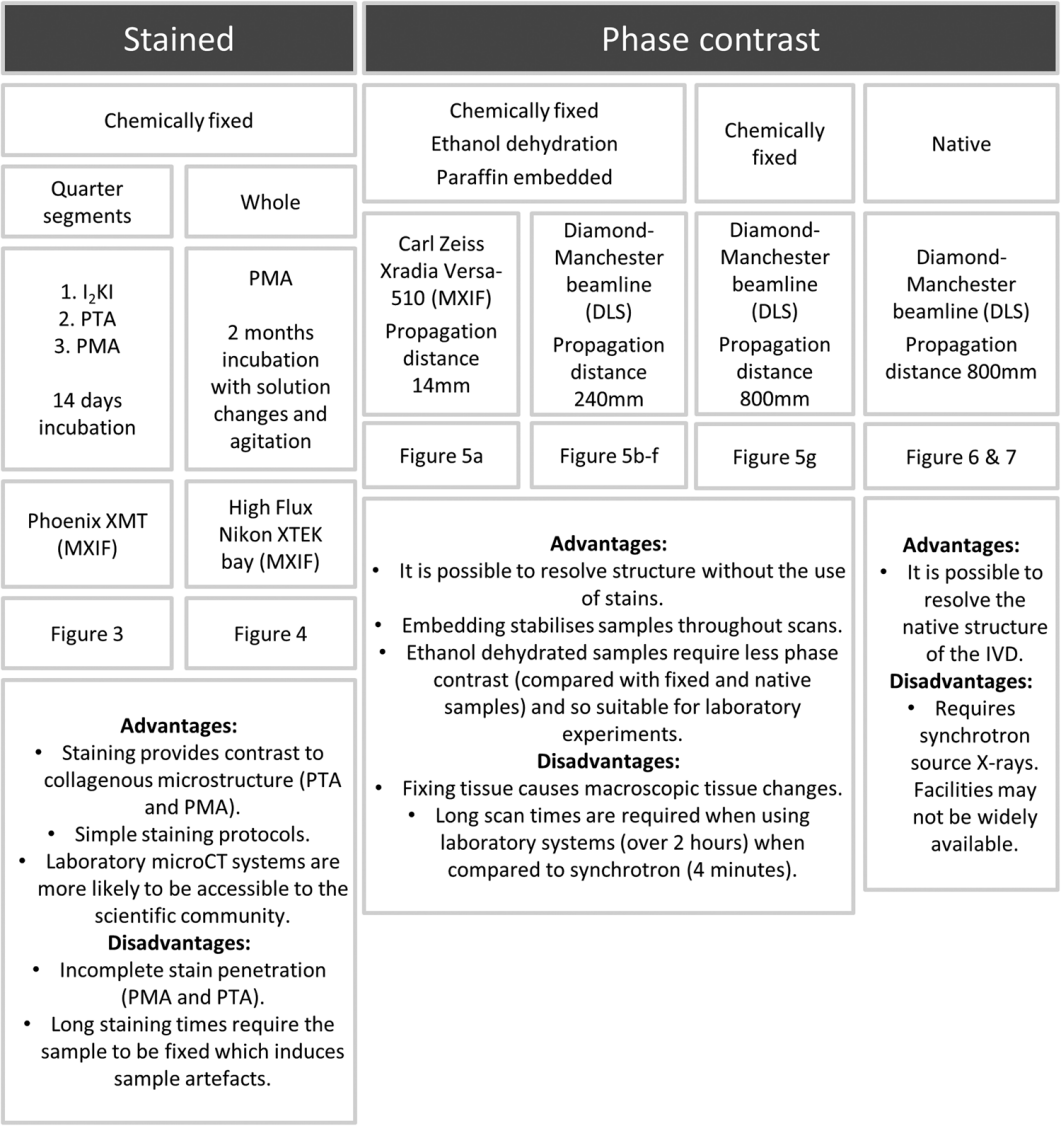
Summary of methods.

The experimental design and biological sample details are summarised in Fig. 2. This figure also conveys the key advantages and disadvantages of different experimental approaches.

### Laboratory microCT of stained IVD segments

**Figure 3:**
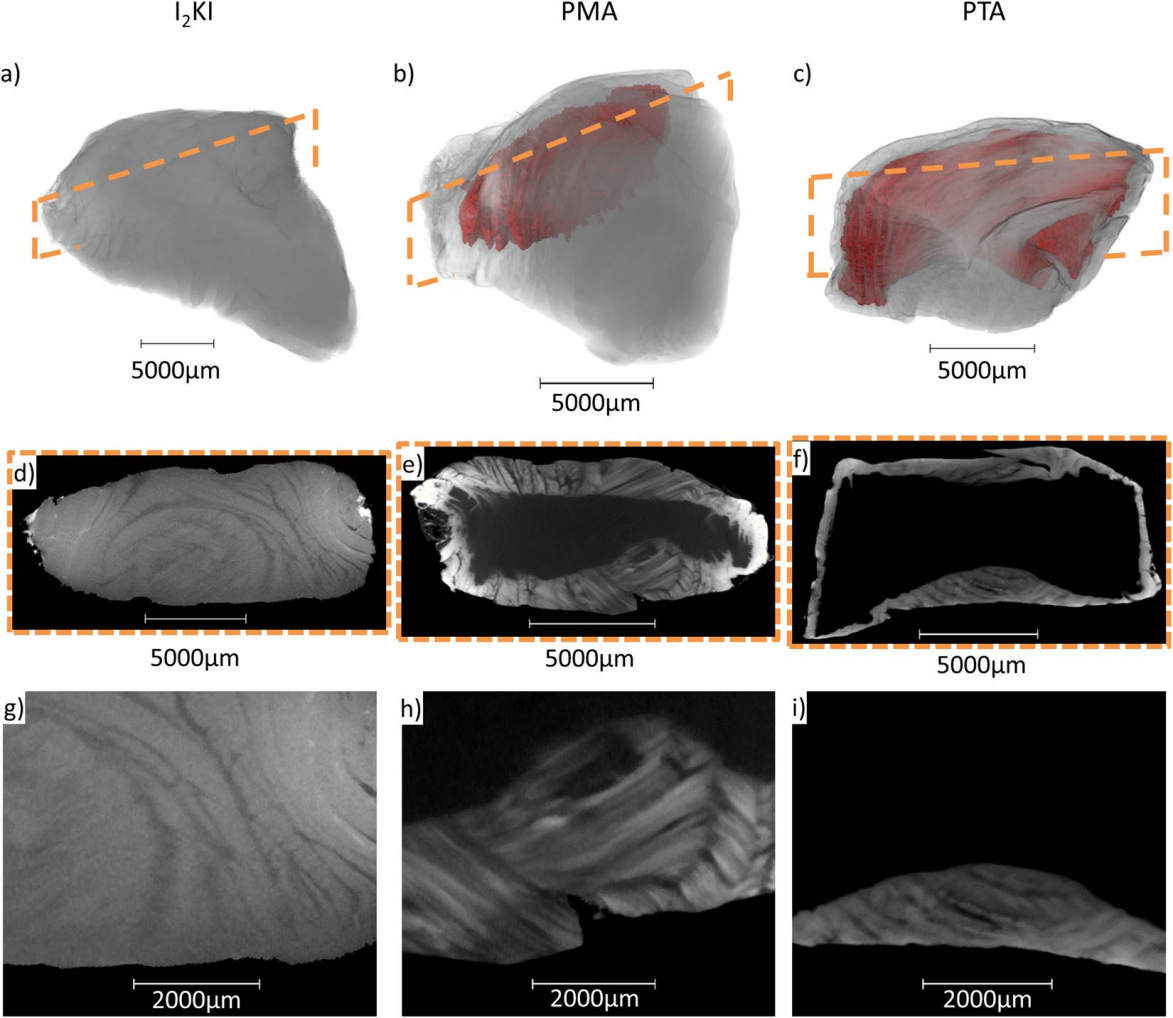
Quarter fixed and stained bovine IVD segments. Three heavy metal stains, I_2_KI (**a**,**d**,**g**), PMA (**b**,**e**,**h**) and PTA (**c**,**f**,**i**), have been evaluated for enhancing contrast after 2 weeks incubation. Tomogram renders show unstained regions in red (**a**–**c**). Radial slices (**d**–**f**) showing large unstained regions using PMA and PTA (**e**,**f**). Magnified radial slices (**g**–**i**) show oblique collagen arrangement in the AF. I_2_KI only provides enough contrast to resolve lamella structures in the outer AF region whereas PMA and PTA allow some internal structure of collagen bundles to be resolved.

Before attempting to stain a whole disc, quarter segments of bovine tail IVD were stained with either iodine potassium iodide (I_2_KI), phosphomolybdic acid (PMA) or phosphotungstic acid (PTA) and scanned on a Phoenix XMT laboratory system. Relative stain penetration and contrast enhancement differed markedly between the three stains after 14 days of incubation (Fig. 3). I_2_KI fully penetrated the segment whilst PMA failed to penetrate the whole AF and PTA staining was largely confined to the segment edges (unstained regions shown by red volumes in Fig. 3b & c). The unstained volume fraction for PMA and PTA segments were 15% and 49% respectively. Radial slices clearly demonstrate that all three stains differentially contrasted AF lamellae (Fig. 3d–f) but only PMA and PTA were able to resolve the alternating arrangement of collagen fibril bundles in the adjacent lamellae (Fig. 3h & i). As a consequence, and given its ability to penetrate the tissues more rapidly than PTA, PMA was chosen to stain intact discs.

### Laboratory microCT of whole IVD stained with PMA

**Figure 4:**
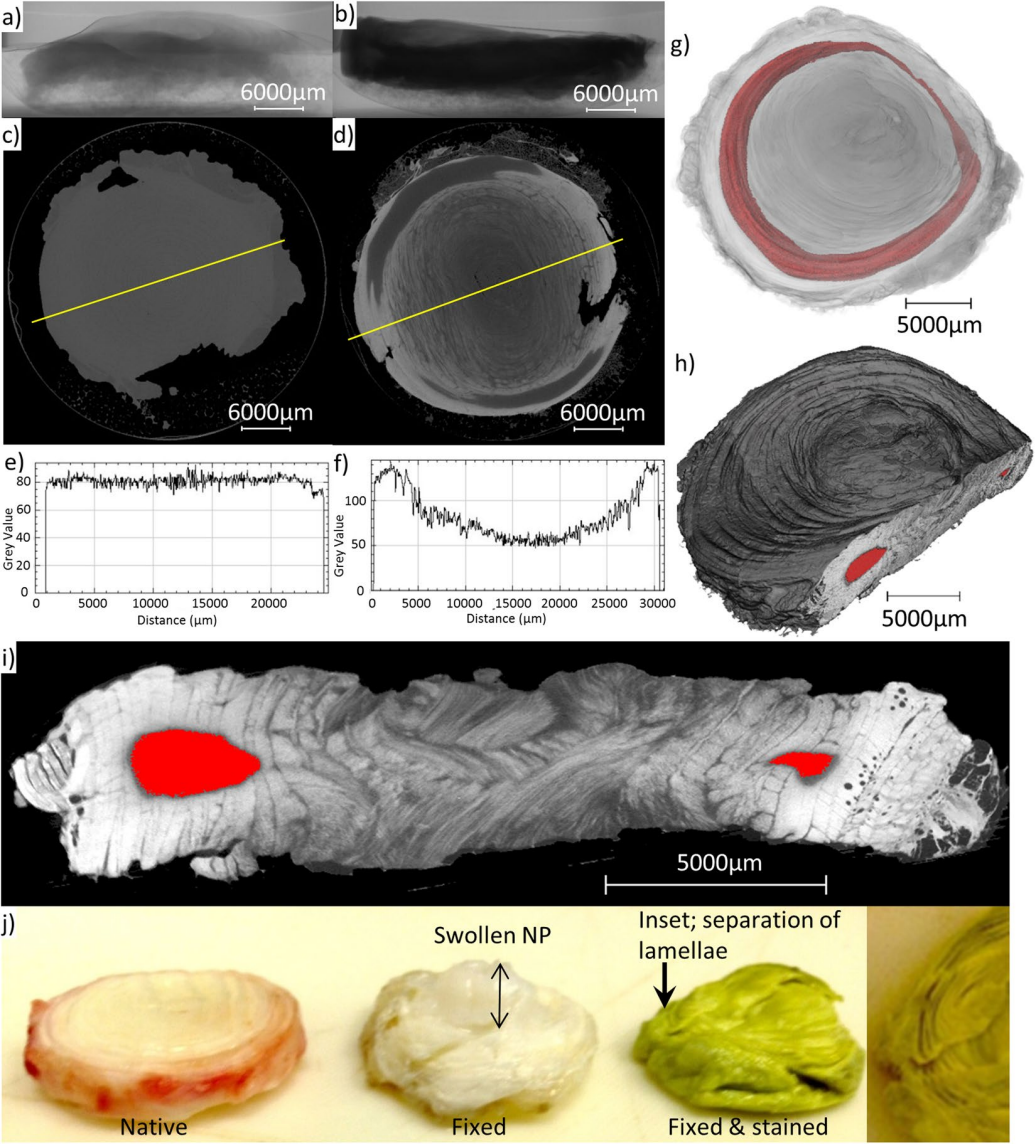
Fixing and staining with PMA increases X-ray contrast but fails to fully penetrate a whole bovine disc and causes major structural changes. Whole intervertebral disc unstained (**a**,**c**,**e**) and stained with PMA for 2 months (**b**,**d**,**f**). Radiograph (**a**), tomography slice (**c**) and associated line profile (**e**) show that X-ray attenuation is low and no structural features were resolved in unstained IVDs. Attenuation and contrast are improved with PMA staining (**b**,**d**,**f**) although there are still some unstained regions shown in red (**g**–**i**). Rendered tomogram (**g**) clipped to show radial slice (**i**). Fixing and staining caused visible structural changes (**j**).

Whole discs were stained with PMA and scanned on High Flux Nikon XTEK bay laboratory system. When compared with an unstained IVD, PMA staining substantially improved radiographic contrast across the whole disc (Fig. 4a & b). As with the quarter disc segments, AF lamellae are clearly visible in transverse slices from tomography volumes of PMA-stained disc. These structures are not discernible in the tomogram of the unstained disc (Fig. 4c & d). Although PMA differentially enhanced contrast of the outer collagenous AF compared with the aggrecan-rich NP (Fig. 4e,f) the stain was unable to penetrate the central region of the AF (Fig. 4g–i), leaving 4% of the disc’s volume unstained. Macroscopically, a comparison of native, chemically fixed and chemically fixed and stained discs showed that fixation and staining caused major structural changes (Fig. 4j). Chemically fixing the disc caused the NP to swell whilst subsequent staining for long incubation times caused AF lamellae to separate. Given the issues with stain penetration and the effects on tissue structure we next imaged unstained but chemically-fixed tissue using phase contrast on both laboratory and synchrotron X-ray sources.

### Laboratory and synchrotron source in-line phase contrast microCT of chemically fixed tissue segments

**Figure 5:**
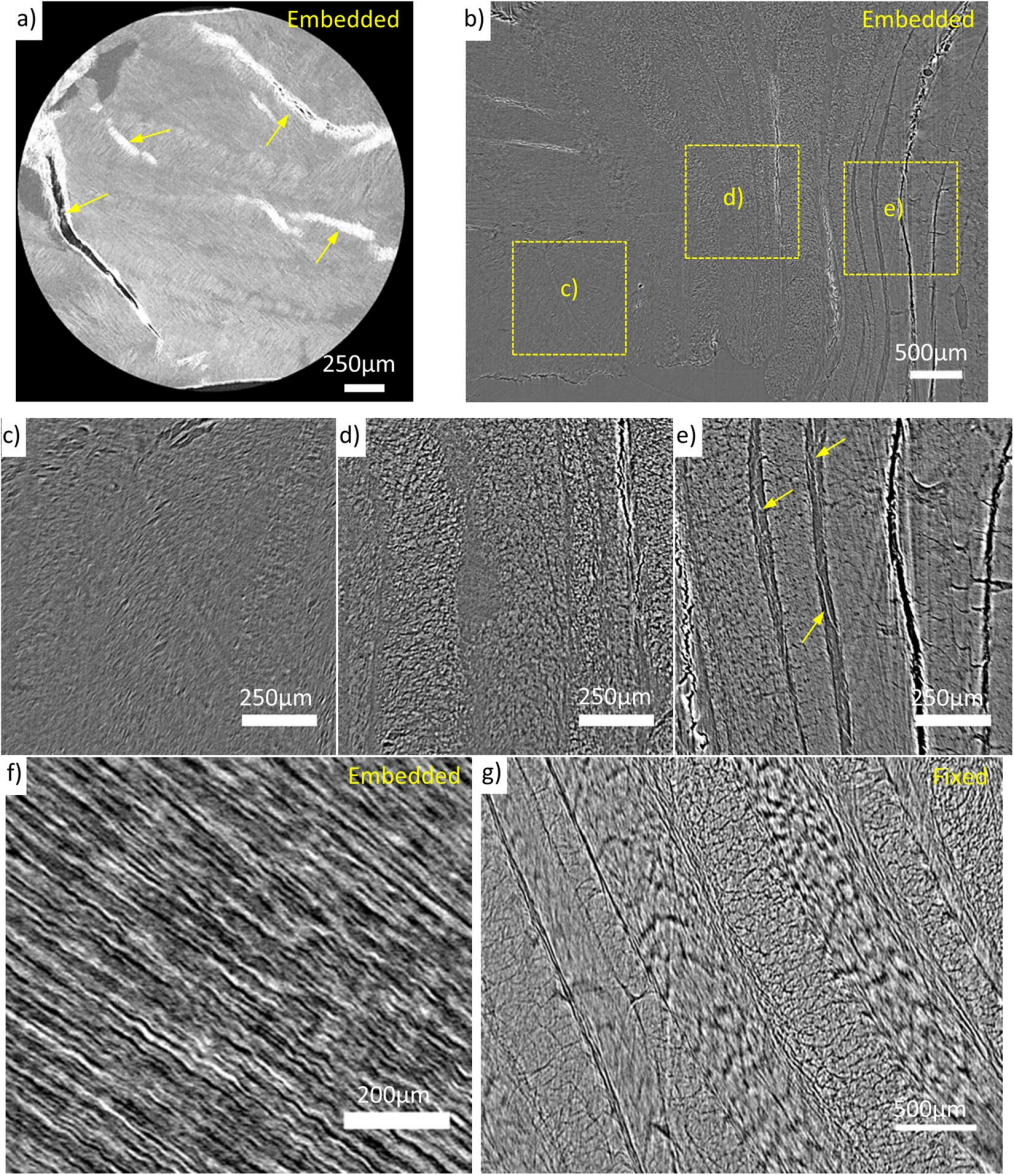
In-line phase contrast microCT of unstained bovine IVD segments. Samples scanned using Carl Zeiss Xradia Versa (MXIF) (**a**) and synchrotron (**b**–**g**). Samples have either been fixed, through an ethanol dehydration series and embedded in paraffin wax (**a**–**f**), or fixed only (**g**). Overview of IVD structure (**c**–**e**) using images taken from one slice (**b**). Collagen fibres (**f**) and AF lamella structure (**g**) were resolved.

We have previously used phase contrast imaging to resolve key structural features in unstained arteries and skin^32^. Using the same laboratory X-ray source (Carl Zeiss Xradia Versa-510, MXIF) it was possible to resolve alternately oriented collagen fibril rich lamella in the AF (Fig. 5a) however the signal to noise ratio was relatively low and cracks in the paraffin resulted in conspicuous artefacts. In order to improve both phase contrast and the signal to noise ratio we next imaged the same samples using higher-flux, higher-coherence synchrotron radiation at beamline I13-2 of Diamond Light Source (DLS). IVD structures resolved in a single slice from the synchrotron tomogram are shown in Fig. 5b–e. The NP has an amorphous structure with extremely fine fibres observed in Fig. 5c. The inner AF has more transitional and less dense lamella structure whereas the outer AF has marked boundaries for each lamella with bridging fibres in the inter-lamella space (indicated by arrows). Taking a radial slice reveals that individual collagen fibres (~5 μm width) have been resolved (Fig. 5f).

Conventional histological tissue processing utilises three stages: ethanol dehydration, chemical fixation and paraffin embedding. We next demonstrated that it was possible to resolve structures, using synchrotron source X-rays, in chemically-fixed tissue without ethanol dehydration or embedding by increasing the amount of phase contrast. The propagation distance was increased from 240 mm for embedded samples to 800 mm for chemically-fixed tissue. The resolved lamellar AF structure is shown in Fig. 5g.

### Synchrotron source in-line phase contrast microCT of native tissue segments

**Figure 6:**
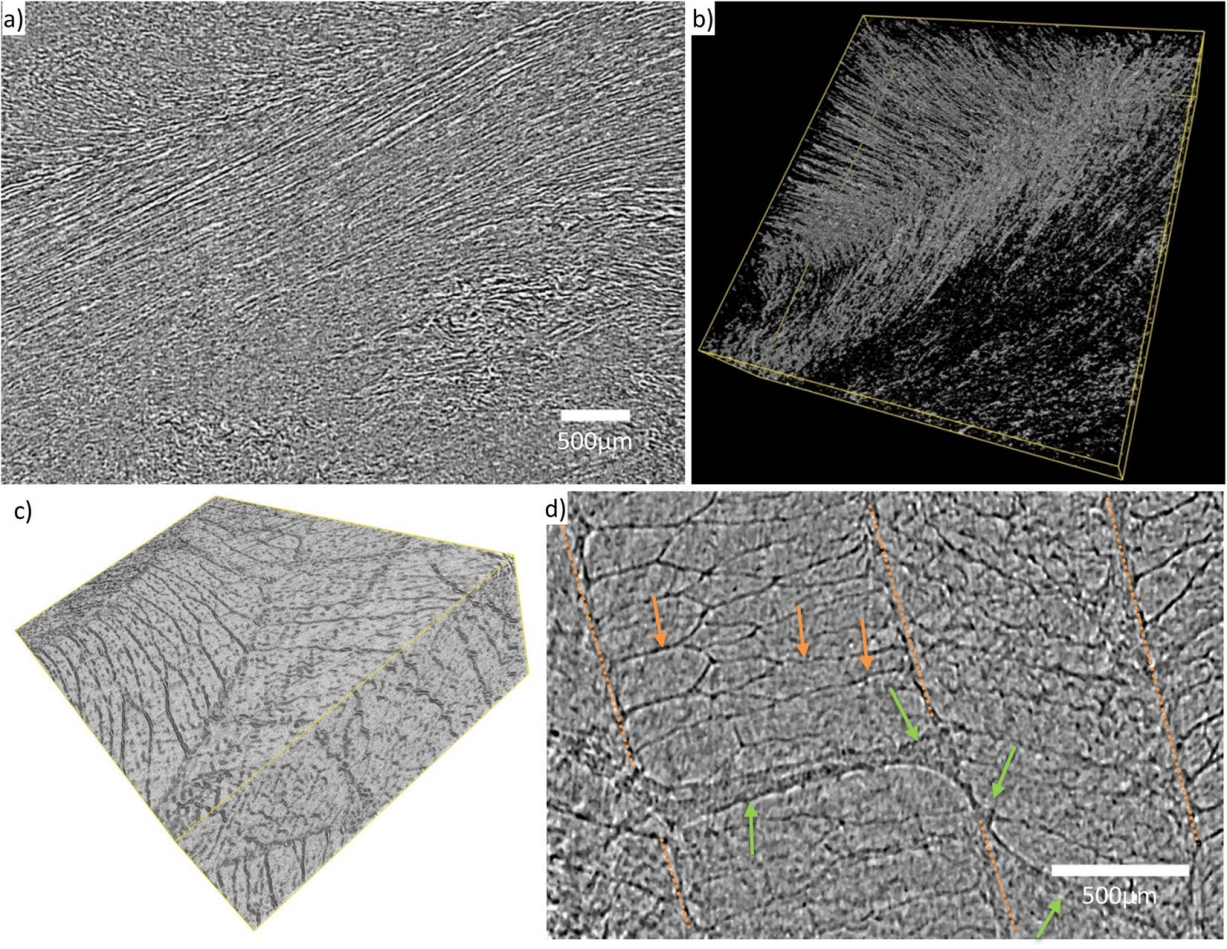
Synchrotron source in-line phase contrast microCT resolves bovine IVD microstructure. Scans taken at I13-2 are capable of resolving native tissue structure at histological resolution. A single slice shows AF collagen bundle alignment (**a**) with 3D render of same region (**b**). Elastic network of AF (volume size panel b: 4500 × 1500 × 160 μm) (**c**,**d**) where orange arrows indicate compartments and green arrows show inter-lamella bridging (volume size panel c: 2540 × 1400 × 500 μm).

Although it is possible to image the microstructure of the unstained IVD chemical fixation of the disc causes structural changes, as seen in Fig. 4j. We therefore next aimed to image native (i.e. non-fixed) tissue. Fine structural details such as collagen bundles, lamella compartments and bridging structures are resolved from the synchrotron source native tissue scans. The images in Fig. 6 were cropped from the original volume due to artefacts around the periphery of the data. A slice and 3D render of the resolved collagen bundles are shown in Fig. 6a & b. The AF elastic network of compartments and bridges is displayed as a volume and slice in Fig. 6c & d. These results show that it is possible to image microstructure in native tissues and visualise slices at any location or angle in the volume. The resolution is slightly reduced due to deformation artefacts (slow tissue relaxation during the scan) but this is offset by the absence of sample preparation (fixation and staining) artefacts.

## Discussion

The quarter segments scanned on the Phoenix XMT laboratory system after two weeks of incubation showed I_2_KI fully stained the IVD tissue but PMA and PTA stains had not completely penetrated the samples. The red rendering in Fig. 3b and c shows that 15% and 49% of the disc’s volume unstained for PMA and PTA-stained segments. Slow or incomplete PMA and PTA penetration is in agreement with other staining studies^26,28,35^. PMA and PTA both have larger molecules (H_3_PMo_12_O_40_ and H_3_PW_12_O_40_) than potassium iodide (KI) and so have lower diffusivity through the dense AF collagen structure. Unstained regions in the PTA and PMA samples were located in the outer AF, as shown by the tomograms (Fig. 3b,c).

I_2_KI staining increased overall X-ray absorption but was limited in revealing tissue sub-structure. This suggests it is more suitable for larger samples such as mammalian organs or smaller organisms where overall anatomical structure is being studied^24,36^. Only the outer AF lamella structure was resolved using I_2_KI whereas both PTA and PMA clearly stained internal lamellae within the AF (Fig. 3g–i). An oblique collagen arrangement is visible in samples stained with PTA and PMA. There is a higher take-up of PMA and PTA in the collagen rich AF when compared to the NP. These results indicate that PTA and PMA specifically stains collagenous structures, which is consistent with previous observations^25,30,31,35^. Both PMA and PTA are present in Masson’s Trichrome, used for detection of collagen fibres, although their role in this three-stage staining protocol is unclear.

Bovine IVDs are relatively large samples (~30 mm) when compared to those used in previous PMA staining studies (<1 mm) such as iliac veins^30^ and articular cartilage^31^. Considering the discs’ volume it was expected that a longer incubation time was required. Agitation and solution changing for whole IVDs was included to aid stain penetration. Solution changing ensured that there was not compound depletion during the two months incubation period. A similar staining pattern was seen in both the quarter segment and whole disc when using PMA; the edges stained with unstained regions inside the AF. This shows that there is not a particular area of the AF that has low affinity but rather the extent of stain penetration through the dense AF structure. Long incubation times, two months for a whole disc, are required for PMA penetration which leads to shrinkage and changes to native tissue structure. Concentration-dependent shrinkage has been reported whilst using I_2_KI stains with 70% shrinkage of cardiac muscle after 14 days incubation^36^. Visible structural changes during fixation include NP swelling and consequently an increase in transverse surface area and tissue volume. Long incubation times during PMA staining caused dehydration of the disc and subsequent separation of lamellae (Fig. 4j). These structural changes are similar to those described in PMA and PTA staining of tendon where shrinkage of up to 25% was inhomogeneous and caused deformation^35^.

The alternating orientation of collagen bundles in a segment of AF tissue has been visualised in 3D using optical coherence tomography^37,38^. Figure 4i shows the bundle organisation in an intact disc (IVD dissected as a whole structure and stained) which has not been visualised previously. However higher resolution is required to fully resolve individual collagen bundles (~5 μm width), but this is limited by the geometric magnification given by a laboratory source cone beam. Multiple structures can be resolved in a whole disc using stains, but the protocol must be improved to stain the entire disc and minimise sample artefacts due to long incubation times. The staining methods used here are simple and are capable of providing contrast to visualise collagenous microstructure (PTA and PMA); however long staining times require the sample to be fixed and staining induces sample artefacts.

Previous work has shown that samples prepared using standard histological preparation (embedded in paraffin wax) and imaged using laboratory source propagation-based phase contrast have sufficient contrast to resolve microstructures in unstained non-calcified tissue^32^. Figure 5a shows the results gained for IVDs using this approach on a Carl Zeiss Xradia Versa laboratory system. Only a small region of interest from a segment of an IVD can be imaged using a laboratory source due to the magnification required to detect interference fringes. A larger field of view and enhanced contrast was possible using synchrotron X-rays at I13-2 (Fig. 5b, Supplementary data 1). However, cracks formed in the supporting paraffin wax and possible effects of dehydration can be seen where cracks aligned with lamella have formed. Further evidence of this is the increase of inter-lamella space, particularly where the lamellae separate and buckle inwards in the outer AF (Fig. 5e). Imaging fixed tissue resulted in fewer of these artefacts but has been shown to cause morphological changes in IVDs (Fig. 4j) and structural changes in collagenous tissue such as smaller fibril diameters^21,22^. Yet imaging fixed tissue was necessary to be a step closer to imaging native tissue.

To give good phase contrast a larger propagation distance was required for the fixed and fresh samples than for the paraffin samples. This could be related to the hydration of the samples. The paraffin samples had been through an alcohol gradient to dehydrate them before paraffin embedding (standard histological preparation). This leads to larger density gradients across boundaries and thus greater phase contrast. These results agree with Dudak *et al*.^39^ whose work showed that ethanol preservation alone greatly improved contrast.

Importantly, we have demonstrated that it is possible to image IVD tissue without the use of stains, although the samples were fixed which caused macroscopic tissue changes. Imaging native tissue reduces the possibility of sample preparation induced artefacts.

**Figure 7:**
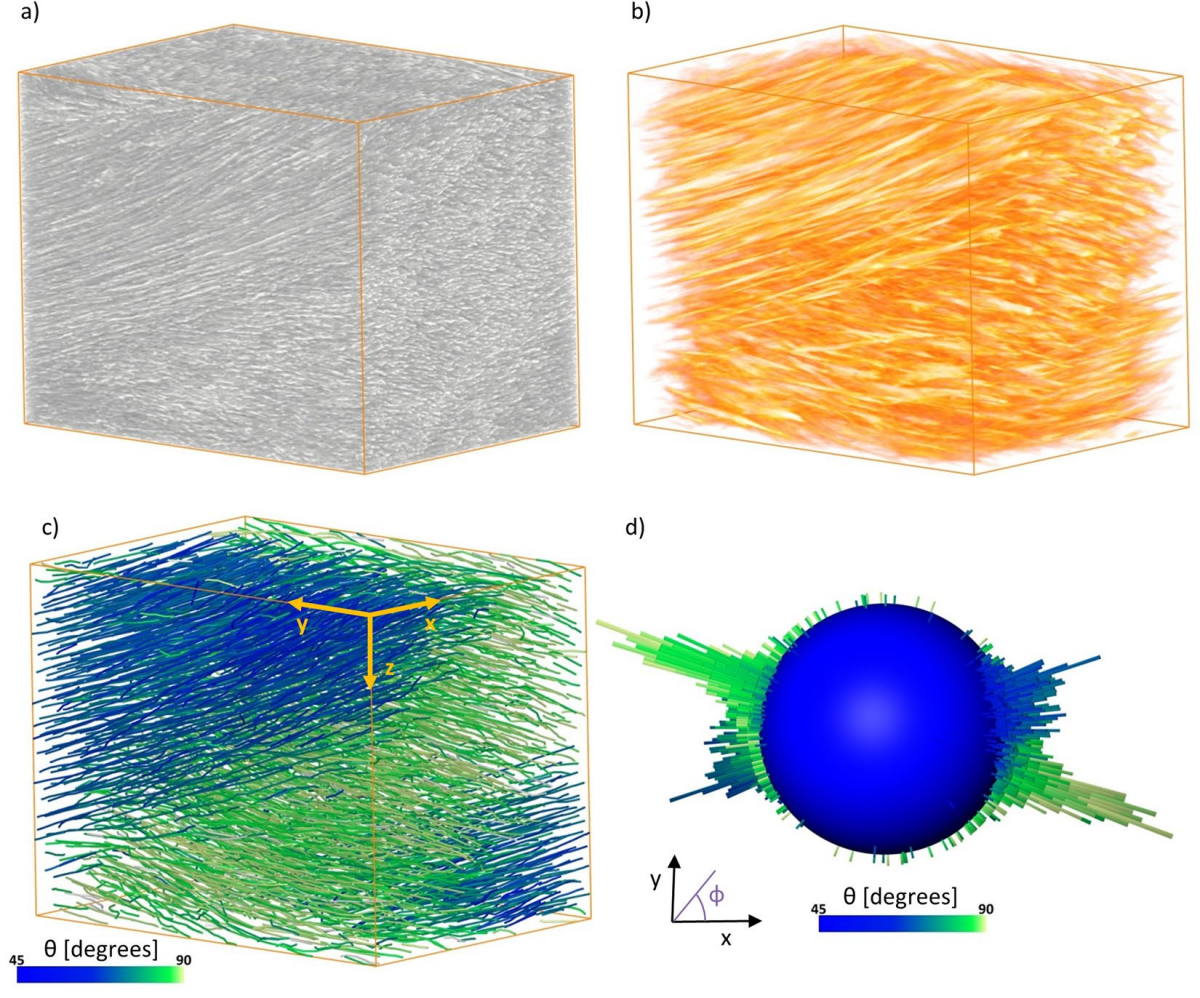
Fibre analysis of native bovine annulus fibrosus. Collagen bundles in a cropped volume (1460 × 1030 × 1140 µm) of AF (**a**) can be correlated to cylinder template (**b**) and individual traced fibres (**c**). The 3D orientation of each fibre is calculated and plotted (**d**). The angle relative to the z axis (θ) is displayed using a colour map and the angle in the x-y plane (ϕ, 0–360) is shown on the orientation sphere (**d**) where bar height is frequency.

Figure 6 demonstrates that it is possible to image native IVD tissue. To our knowledge, only one study has previously imaged the microstructure of native IVD tissue with a laboratory system^40^. Laboratory systems have long scans, for example up to 17 hours (2500 projections at 25 s exposure) reported in Naveh *et al*.^40^. The mouse IVD AF structure is seen to buckle inwards which is suggestive of sample drying during the long scan time, but the authors comment that this may be related to asymmetric loading. Two different types of IVD structure can be visualised in Fig. 6. Firstly (Figs 6a,b and 7a, Supplementary data 2), AF collagen bundles can be seen with a similar resolution to those imaged using confocal techniques^41,42^. Secondly (Fig. 6c,d), lamella structure and compartments can be determined from reconstructed slices, which is comparable to results from DIC imaging^43^ and confocal microscopy^41^.

Reports of the elastic network (AF compartments and bridges) in the literature vary depending on the imaging technique used. Elastic fibres (composed of elastin and fibrillin microfibrils) have been found throughout the IVD with the highest concentration in the interlamelar space and in bridging elements between adjacent lamellae^43,44^. AF tissue sections stained with toluidine blue/fast green reveals the translamellar cross bridges (TLCBs) as seen by Melrose *et al*.^45^. However, when using digital interference contrast of serial sectioned AF^46,47^ or optical coherence tomography of mesoscale volumetric section of AF^37^, the structural complexity was far greater than previously thought from 2D histological studies. Therefore it is critical to image these structures in 3D in their native form. Using microCT, elastic fibres similar to those seen by Naveh *et al*.^40^, crossing the inter-lamella space in the paraffin-embedded samples have been identified (Fig. 5e). These structures cannot be seen in the fixed and fresh tissue scans; instead the lamellae are closely packed with less organised material between. It may be suggested that dehydration (by paraffin embedding or by the long scan time utilised by Naveh *et al*.^40^) causes buckling and separation of the lamellae which in turn causes the fibres in the elastic network to be stretched and made visible. Large and small trans-lamella bridges and the finer elastic network within the lamella compartments have been resolved (Fig. 6c,d). This shows that the elastic network is a multiscale and a fully integrated structure as demonstrated by Yu *et al*.^43^ using immunostaining.

Fresh tissue is ideal as it possesses near native structure and mechanics, but there may be some imaging artefacts due to sample movement. Artefacts due to sample movement can be minimised by fast scan times, stabilising using a load^40^ and by allowing the tissue to relax before scanning^48^. Region of interest scanning of a relatively large sample also produces artefacts. The displayed images have been cropped to displayed useable data. A further limitation of taking a region of interest scan is that there is some uncertainty of the exact anatomical location the scan relates to. Future studies may consider a smaller animal model which is more suited to the field of view available with the 1.6 μm effective pixel size.

Not only is there a need to visualise the 3D native tissue structure but also to use the acquired image data to quantify structures. Figure 7 gives an example of how microCT data can be used to quantify the collagen bundle structure of the AF. Individual bundles can be extracted from the image data and analysed in 3D (Fig. 7b,c). Bundle orientation is plotted (Fig. 7d) and shows the directional grouping from alternate lamellae.

MicroCT is capable of providing 3D images of intact samples which can be used for structural characterisation of the IVD. Figure 2 lists advantages and disadvantages of each approach used in this study. Simple staining protocols using PMA were found to stain the collagenous structure of the AF although it is challenging to fully stain whole bovine discs and staining led to visual changes in structure. Nevertheless, laboratory CT approaches used in this study have the potential to quantify changes in tissue structure and mechanisms of AF degeneration which are still unidentified. Importantly, it was possible to resolve a high level of detail in fresh tissue using synchrotron radiation and propagation-based phase contrast. It should be noted that access to synchrotron microCT is limited and not likely to be as available as a laboratory microCT system. Imaging whole fresh IVDs means that native structure and mechanics are preserved which is significant for potential 3D structural mechanical studies at resolutions in the micron range. These methods have the potential to characterise age-related structural remodelling in cartilaginous tissues. This has particular significance for the IVD as both an understanding of structural disc mechanics during progressive degeneration and efficacy assessment of tissue replacements are required for successful LBP treatments.

## Methods

### Tissues and materials

Three bovine tails (age range 18–36 months) were obtained from the local abattoir (Kurpas Meats PLC). IVDs were dissected as whole structures without endplates. Whole disc samples were ~3 cm in diameter and 1 cm in height. Sample processing and imaging are detailed below with a summary in Fig. 2. Three stains were chosen for comparison: iodine potassium iodide (I_2_KI), phosphomolybdic acid (PMA) and phosphotungstic acid (PTA). All stains were purchased from Sigma Aldrich and solutions were prepared following a protocol based on Metscher *et al*.^27^. A stock of I_2_KI was prepared by adding (1 g per l) iodine metal (I_2_) and (2 g per l) potassium iodide (KI) in water and diluting to 10% (v/v) in water just before use. PMA and PTA were made to the same concentration of (2 g per l) in water and diluted to 30% (v/v) in absolute ethanol. Due to PMA photosensitivity, the solution was stored and used in opaque containers.

### MicroCT of chemically-fixed and stained disc segments and whole discs

Whole IVDs were immediately chemically fixed in 10% formal saline for 24 hours at room temperature and divided into quarter segments to compare the three stains for contrast and penetration. The segments were rinsed with phosphate buffered saline (PBS) before being placed in the staining solutions. After 14 days incubation the segments were then rinsed in 70% ethanol, wrapped in parafilm to minimise drying and scanned in a plastic tube. The segments were scanned using the Phoenix XMT system at the Manchester X-ray Imaging Facility (MXIF: Diamond-Manchester Collaboration, Research Complex at Harwell). The scanner settings were set to a source voltage between 60–90 kV. 2001 projections of 1 s exposure time were recorded. Reconstruction software (Phoenix dato s|x2 reconstruction) was used to generate 3D dataset from the projections. The effective voxel size for the segments varied between 19–21 μm.

Given the low diffusion rate for quarter segments, whole IVDs were incubated in PMA solution for 2 months with regular solution changes. Samples were left at room temperature with gentle agitation to aid stain penetration. The discs were rinsed in 70% ethanol and placed in a membrane box with a biopsy pad (Fig. 1b). The membrane clamped the sample to provide stability during the scan. The High Flux Nikon XTEK bay at MXIF was used to image whole discs at source voltage 100 kV (15.8 W). 6433 projections of 1 s exposure time were recorded. Reconstruction software (Nikon 3D pro) was used to give a theoretical reconstructed voxel size of 8.2 μm. Reconstruction of all the stained samples included a beam-hardening correction (Nikon 3D pro). Avizo 8.0 was used to visualise and process the reconstructed data. A median filter was applied to denoise the images and a watershed segmentation algorithm was used to separate the sample from the holder. A volume fraction of unstained:stained tissue was calculated for PTA and PMA samples using number of voxels in the segmented volume.

### Laboratory and synchrotron phase contrast microCT of unstained IVD tissue

Unstained samples were initially imaged using a laboratory-based microCT system Carl Zeiss Xradia Versa at MXIF with in-line phase contrast enhancement. The sample preparation and imaging were based on methods published by Walton *et al*.^32^. For mounting the samples in paraffin, the fixed IVDs were dehydrated through an alcohol gradient and set in paraffin using Thermo Shandon Citadel 2000 and Thermo Shandon Histocentre 3. A ~3 mm tissue segment of AF was taken to match the system’s field of view and excess paraffin was trimmed before scanning. The source voltage was set to 90 kV (8 W) to provide contrast in the sample and 2501 projections were recorded over 360° using a 4x objective with 3 s exposure time. Source-to-sample and sample-to-detector distances were 33 and 14 mm respectively. This propagation distance (sample to detector) allowed for a small amount of phase contrast sufficient to resolve the AF microstructure. The projections were reconstructed using Xradia Versa Reconstructor to achieve an effective voxel size of 2.8 µm.

Paraffin embedded samples were also scanned on the Diamond-Manchester Imaging Branchline I13-2 at Diamond Light Source using a filtered (1.3 mm pyrolytic graphite, 3.2 mm aluminium and 70 µm steel) pink beam (5–35 keV) with an undulator gap of 5 mm. The higher flux, higher coherence and scope for large propagation distances at I13-2 allows for quicker imaging with greater phase contrast when compared to a laboratory system. An ~8 mm tissue segment (including NP and AF) was chosen for this study to fit the field of view. The sample was wrapped in film and contained in a sealed plastic tube to minimise drying during the scan. Sample alignment in the beam was under low dose conditions (large undulator gap and use of shutters). Projections were recorded using the pco.edge 5.5 scintillator-coupled detector (2560 × 2160 pixels and a physical pixel size of 6.5 µm). A 2x objective lens was used to achieve a total magnification of 4x, an effective pixel size of 1.6 µm and a field of view of 4.2 × 3.5 mm. A 4x objective lens was used to achieve a total magnification of 8x, an effective pixel size of 0.81 µm and a field of view of 2.1 × 1.8 mm. Exposure times of 0.045 s and 0.06 s for 4x and 8x total magnification were chosen to give counts representing ~50% of saturation in flat-field images (without sample in beam path). A total of 4001 projection images was found to provide a good compromise between signal:noise and tissue-relaxation artefacts; these were recorded over 180° of continuous rotation (‘fly scan’) and reconstructed using the proprietary DLS software DAWN^49,50^. The propagation distance was increased in ~200 mm increments until sufficient in-line phase contrast was gained to visualise the microstructures. For the sample embedded in paraffin the propagation distance was 240 mm, whereas 800 mm was required for the fixed and native samples. Avizo XFiber Extension^51,52^ was used to extract and analyse the collagen bundle structure in the AF. Cylinder Correlation was used to enhance the collagen bundle (fibre-like) structures (Fig. 7b). The correlation lines were then traced and displayed as fibres (Fig. 7c). Orientation is given by phi (φ, x-y plane relative to the x axis) and theta (θ, with respect to the z axis). Fibre 3D orientation can either be displayed using a coloured fibre render (Fig. 7c) or orientation sphere (Fig. 7d).

### Data availability

A representative sample of the stained data is provided in supplementary material [doi: 10.17632/x97 × 865jpm.1]. Other datasets generated during and/or analysed during this study are not publicly available due to their large size but are available from the corresponding author on reasonable request.

## Electronic supplementary material

Supplementary video 1

Supplementary video 2
